# *N*-(3-Oxo-1-thia-4-aza­spiro­[4.5]dec-4-yl)-6-phenyl­imidazo[2,1-*b*][1,3]thia­zole-3-acetamide hemihydrate

**DOI:** 10.1107/S1600536808009306

**Published:** 2008-04-10

**Authors:** Mehmet Akkurt, Şerife Pınar Yalçın, Nuray Ulusoy Güzeldemirci, Orhan Büyükgüngör

**Affiliations:** a Department of Physics, Faculty of Arts and Sciences, Erciyes University, 38039 Kayseri, Turkey; b Department of Pharmaceutical Chemistry, Faculty of Pharmacy, Istanbul University, 34116 Istanbul, Turkey; c Department of Physics, Faculty of Arts and Sciences, Ondokuz Mayıs University, 55139 Samsun, Turkey

## Abstract

The title compound, C_21_H_22_N_4_O_2_S_2_·0.5H_2_O, crystallizes with two mol­ecules in the asymmetric unit. The dihedral angles between the phenyl and imidazothiazole ring systems are 19.16 (9) and 21.37 (9)°. In the imidazothiazole ring systems, the cyclohexane rings adopt chair conformations, while the thiazole rings have distorted envelope conformations. The two mol­ecules are stabilized by intra­molecular N—H⋯O, O—H⋯O and C—H⋯S inter­actions and the crystal structure is stabilized by inter­molecular N—H⋯O, O—H⋯O, C—H⋯O and C—H⋯N inter­actions.

## Related literature

For related literature, see: Akkurt *et al.* (2005 ▶, 2007 ▶); Allen *et al.* (1987 ▶); Amarouch *et al.* (1988 ▶); Andreani *et al.* (1998 ▶); Cremer & Pople (1975 ▶); Devlin & Hargrave (1989 ▶); Gürsoy & Ulusoy Güzeldemirci (2007 ▶); Srimanth *et al.* (2002 ▶); Ulusoy (2002 ▶); Ur *et al.* (2004 ▶); Öztürk Yıldırım, Akkurt, Ur, Cesur, Cesur & Büyükgüngör (2005 ▶); Öztürk Yıldırım, Akkurt, Ur, Cesur, Cesur & Heinemann (2005 ▶).
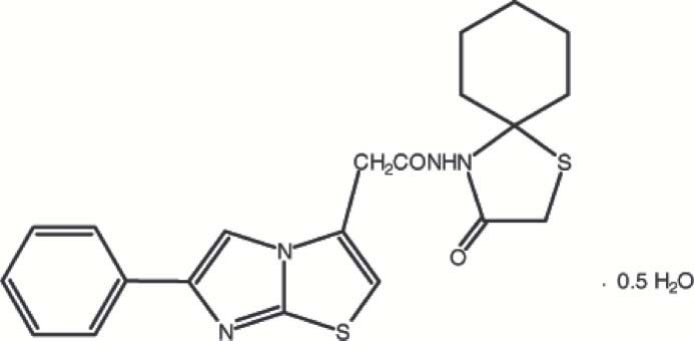


## Experimental

### 

#### Crystal data

C_21_H_22_N_4_O_2_S_2_·0.5H_2_O
*M* *_r_* = 435.58
Triclinic, 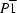

*a* = 11.0175 (3) Å
*b* = 11.8817 (3) Å
*c* = 17.6162 (5) Å
α = 75.123 (2)°
β = 73.502 (2)°
γ = 81.012 (2)°
*V* = 2128.36 (10) Å^3^
*Z* = 4
Mo *K*α radiation
μ = 0.28 mm^−1^
*T* = 296 K
0.52 × 0.39 × 0.25 mm

#### Data collection

Stoe IPDSII diffractometer
Absorption correction: integration (*X-RED32*; Stoe & Cie, 2002 ▶) *T*_min_ = 0.869, *T*_max_ = 0.934
38012 measured reflections
8351 independent reflections
6705 reflections with *I* > 2σ(*I*)
*R*_int_ = 0.054

#### Refinement

*R*[*F* ^2^ > 2σ(*F* ^2^)] = 0.039
*wR*(*F* ^2^) = 0.098
*S* = 1.02
8351 reflections
538 parameters
3 restraints
H atoms treated by a mixture of independent and constrained refinement
Δρ_max_ = 0.29 e Å^−3^
Δρ_min_ = −0.34 e Å^−3^

### 

Data collection: *X-AREA* (Stoe & Cie, 2002 ▶); cell refinement: *X-AREA*; data reduction: *X-RED32* (Stoe & Cie, 2002 ▶); program(s) used to solve structure: *SIR97* (Altomare *et al.*, 1999 ▶); program(s) used to refine structure: *SHELXL97* (Sheldrick, 2008 ▶); molecular graphics: *ORTEP-3 for Windows* (Farrugia, 1997 ▶); software used to prepare material for publication: *WinGX* (Farrugia, 1999 ▶).

## Supplementary Material

Crystal structure: contains datablocks global, I. DOI: 10.1107/S1600536808009306/hg2390sup1.cif

Structure factors: contains datablocks I. DOI: 10.1107/S1600536808009306/hg2390Isup2.hkl

Additional supplementary materials:  crystallographic information; 3D view; checkCIF report

## Figures and Tables

**Table 1:** Hydrogen-bond geometry (Å, °)

*D*—H⋯*A*	*D*—H	H⋯*A*	*D*⋯*A*	*D*—H⋯*A*
N3—H3*A*⋯O3	0.86	1.98	2.841 (2)	175
O5—H5*A*⋯O1	0.833 (19)	1.880 (19)	2.7097 (19)	174.4 (18)
O5—H5*B*⋯O4^i^	0.848 (18)	1.917 (18)	2.764 (2)	177 (2)
N7—H7⋯O5^ii^	0.86	1.94	2.7617 (19)	160
C10—H10⋯O4^i^	0.93	2.41	3.306 (2)	161
C12—H12*A*⋯O2^i^	0.97	2.48	3.074 (2)	120
C15—H15*B*⋯N2^iii^	0.97	2.57	3.462 (3)	153
C18—H18*A*⋯S2	0.97	2.87	3.255 (2)	105
C20—H20*B*⋯S2	0.97	2.84	3.227 (3)	105
C21—H21*A*⋯S4	0.97	2.83	3.768 (2)	163
C33—H33*B*⋯O5^ii^	0.97	2.54	3.376 (2)	144
C36—H36*B*⋯N6^iii^	0.97	2.56	3.448 (3)	153
C39—H39*A*⋯S4	0.97	2.87	3.246 (2)	104
C41—H41*B*⋯S4	0.97	2.78	3.194 (2)	106

Symmetry codes: (i) 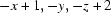; (ii) 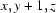; (iii) 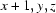.

**Fig. 1.**
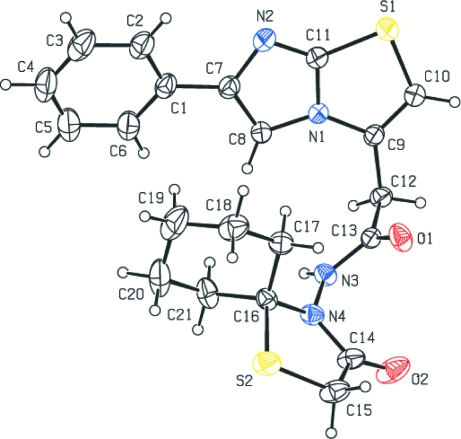
An ORTEP-3 plot of the molecule A in the asymmetric unit, with the atom numbering scheme. Displacement ellipsoids for non-H atoms are drawn at the 30% probability level.

**Fig. 2.**
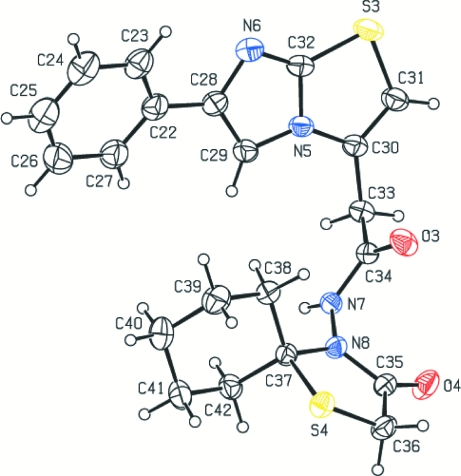
An ORTEP-3 plot of the molecule Bs in the asymmetric unit, with the atom numbering scheme. Displacement ellipsoids for non-H atoms are drawn at the 30% probability level.

**Fig. 3.**
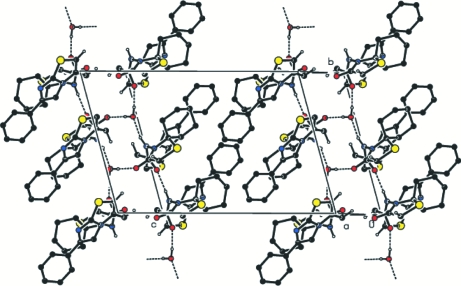
View of the packing and hydrogen bonding interactions for the title compound in the unitcell.

**Fig. 4.**
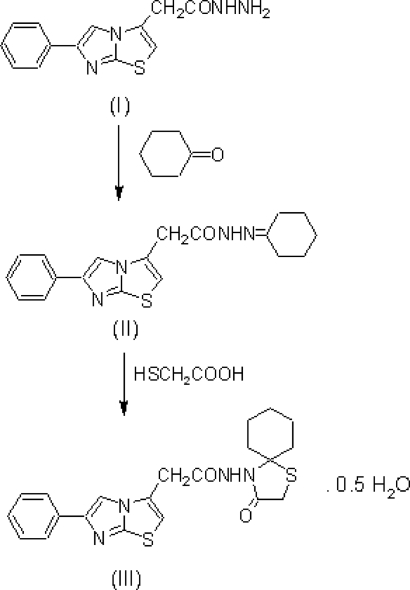
The formation of the title compound.

## supplementary crystallographic information

### Comment

Imidazo[2,1-*b*]thiazolederivatives have demonstrated a broad range of biological activities, including immunoregulatory (Devlin & Hargrave, 1989), anticancer (Srimanth *et al.*, 2002), antihelmintic (Amarouch *et al.*, 1988), cardiotonic (Andreani *et al.*, 1998) and antimycobacterial (Ur *et al.*, 2004). In connection with our previous papers on the synthesis of imidazo[2,1-*b*]thiazoles (Gürsoy & Ulusoy Güzeldemirci, 2007) and their crystal structures (Akkurt *et al.*, 2007), we report here the crystal structure of the title spiro derivative, 6-phenyl-*N*-(3-oxo-1-thia-4-azaspiro[4.5]dec-4-yl)-imidazo[2,1-\ *b*]thiazole-3-acetamide hemihydrate (III) (Scheme 1 and 2).

In the two molecules A (Fig. 1) and B (Fig. 2) of the title compound, the bond lengths in two molecules are normal (Allen *et al.*, 1987). The mean C—S bond length [1.778 (2) Å] in two molecules may be compared with the corresponding values in similar molecules [1.737 (5) Å (Akkurt *et al.*, 2007), 1.7588 (2) Å (Öztürk Yıldırım, Akkurt, Ur, Cesur, Cesur & Büyükgüngör, 2005), 1.783 (2) Å (Öztürk Yıldırım, Akkurt, Ur, Cesur, Cesur & Heinemann, 2005) and 1.729 (2) Å (Akkurt *et al.*, 2005)].

The thiazole and imidazole rings in the two molecules A [with S1] and B [with S3] of the title compound are essentially coplanar, with a dihedral angle of 2.29 (10) and 1.33 (10)°, respectively. The dihedral angles of the benzene rings and the mean plane of the thiazole and imidazole rings systems are19.16 (9) and 21.37 (9)° for molecules A and B, respectively. The other thiazole rings have distorted envelope conformations [puckering parameters (Cremer & Pople, 1975): Q(2) = 0.1773 (2) Å, φ(2) = 342.0 (6) ° for molecule A, and Q(2) = 0.193 (2) Å, φ(2) = 170.2 (5) ° for molecule B], while the cyclohexane rings to connected to them have chair conformation for two molecules in the asymmetric unit [puckering parameters: Q_T_ = 0.567 (3) Å, θ =178.1 (3) °, φ = 179 (7) ° for molecule A, and Q_T_ = 0.570 (2) Å, θ =179.6 (2) °, φ = 109 (6) ° for molecule B].

The two molecules are stabilized by intramolecular N—H···O, O—H···O and C—H···S interactions and the crystal packing is stabilized by intermolecular N—H···O, O—H···O, C—H···O and C—H..N hydrogen bonding interactions (Table 1, Fig. 3).

### Experimental

A mixture of 6-phenyl-*N*-(cyclohexylidene)imidazo[2,1-*b*]thiazole-3-\ acetohydrazide (0.005 mol) and HSCH_2_COOH (0.01 mol) was refluxed in dry benzene (30 ml) using a Dean-Stark trap for 48 h. Excess benzene was evaporated *in vacuo*.The residue was triturated with saturated NaHCO_3_ until CO_2_ evolution ceased and then allowed to stand overnight. The solid thus obtained was filtered, washed with H_2_O and recrystallized from C_2_H_5_OH to yield colourless prisms of (III) (Ulusoy, 2002).

IR [ν, cm^-1^,KBr]: 3390, 3272 (O—H, N—H), 1718,1670 (C=O). ^1^H-NMR [δ, p.p.m.,DMSO-*d_6_*]: 0.92–1.78 (10*H*, m, cyclohex.), 3.60 (2*H*, s, thiazolidinone SCH_2_),3.93 (2*H*, s, CH_2_CO), 7.10(1*H*, s, imidazothiazole C_2_—H),7.21–7.43 (3*H*, m, ph.), 7.81 (2*H*, d, J= 7.6 Hz, ph.), 8.23 (1*H*, s, imidazothiazole C_5_—H),10.45 (1*H*, s, CONH). ^13^C-NMR(APT) [δ, p.p.m., DMSO-*d_6_*]: 22.64 (cyclohex. C_4_), 23.70 (cyclohex. C_3_ and C_5_),27.62 (thiazolidinone C_5_), 32.48 (s, CH_2_CO), 36.69 (cyclohex. C_2_ and C_6_),71.91 (thiazolidinone C_2_), 108.15 (imidazothiazole C_5_),110.12 (imidazothiazole C_2_), 124.36 (ph. C_4_), 126.77 (ph.C_3_ and C_5_), 128.40 (ph. C_2_ and C_6_),125.68, 134.08, 145.90, 148.42 (imidazothiazole C_3_, C_6_, C_7a_and ph. C_1_), 166.54 (CONH), 167.64 (thiazolidinone C=O). EI—MS (70 eV), m/*z* (%): 426 (*M*^+^, 70), 353 (72), 257 (9), 241 (69), 214 (100). Analysis calculated for C_21_H_22_N_4_O_2_S_2_.0.5H_2_O: C 57.90, H 5.32, N 12.86%. Found: C 57.87, H 5.77, N 12.94%.

### Refinement

The H atoms of the water molecule were found from a difference Fourier map and refined freely. *DFIX* restraints were applied to the O—H distances [0.83 (2) Å] and H—O—H angles [by restraining the H···H distances to 1.40 (2) Å]. The other H atoms were positioned geometrically, with N—H = 0.86 Å, C—H = 0.93 and 0.97 Å, and refined using a riding model, with *U*_iso_(H) = 1.2*U*_eq_(C,*N*).

### Crystal data

**Table:**

C_21_H_22_N_4_O_2_S_2_·0.5H_2_O	*Z* = 4
*M**_r_* = 435.58	*F*_000_ = 916
Triclinic, *P*1	*D*_x_ = 1.359 Mg m^−^^3^
Hall symbol: -P 1	Mo *K*α radiation λ = 0.71073 Å
*a* = 11.0175 (3) Å	Cell parameters from 51089 reflections
*b* = 11.8817 (3) Å	θ = 1.8–28.0º
*c* = 17.6162 (5) Å	µ = 0.28 mm^−^^1^
α = 75.123 (2)º	*T* = 296 K
β = 73.502 (2)º	Block, colourless
γ = 81.012 (2)º	0.52 × 0.39 × 0.25 mm
*V* = 2128.36 (10) Å^3^	

### Data collection

**Table:**

Stoe IPDS2 diffractometer	8351 independent reflections
Monochromator: plane graphite	6705 reflections with *I* > 2σ(*I*)
Detector resolution: 6.67 pixels mm^-1^	*R*_int_ = 0.054
*T* = 296 K	θ_max_ = 26.0º
ω scans	θ_min_ = 1.8º
Absorption correction: integration(X-RED32; Stoe & Cie, 2002)	*h* = −13→13
*T*_min_ = 0.869, *T*_max_ = 0.934	*k* = −14→14
38012 measured reflections	*l* = −21→21

### Refinement

**Table:**

Refinement on *F*^2^	Secondary atom site location: difference Fourier map
Least-squares matrix: full	Hydrogen site location: inferred from neighbouring sites
*R*[*F*^2^ > 2σ(*F*^2^)] = 0.039	H atoms treated by a mixture of independent and constrained refinement
*wR*(*F*^2^) = 0.098	*w* = 1/[σ^2^(*F*_o_^2^) + (0.0455*P*)^2^ + 0.3604*P*] where *P* = (*F*_o_^2^ + 2*F*_c_^2^)/3
*S* = 1.02	(Δ/σ)_max_ = 0.001
8351 reflections	Δρ_max_ = 0.29 e Å^−^^3^
538 parameters	Δρ_min_ = −0.34 e Å^−^^3^
3 restraints	Extinction correction: none
Primary atom site location: structure-invariant direct methods	

### Special details

**Table:**

Geometry. Bond distances, angles *etc*. have been calculated using the rounded fractional coordinates. All su's are estimated from the variances of the (full) variance-covariance matrix. The cell e.s.d.'s are taken into account in the estimation of distances, angles and torsion angles
Refinement. Refinement on *F*^2^ for ALL reflections except those flagged by the user for potential systematic errors. Weighted *R*-factors *wR* and all goodnesses of fit *S* are based on *F*^2^, conventional *R*-factors *R* are based on *F*, with *F* set to zero for negative *F*^2^. The observed criterion of *F*^2^ > σ(*F*^2^) is used only for calculating -*R*-factor-obs *etc*. and is not relevant to the choice of reflections for refinement. *R*-factors based on *F*^2^ are statistically about twice as large as those based on *F*, and *R*-factors based on ALL data will be even larger.

### Fractional atomic coordinates and isotropic or equivalent isotropic displacement parameters (Å^2^)

**Table:**

	*x*	*y*	*z*	*U*_iso_*/*U*_eq_	
S1	0.05906 (5)	−0.07649 (4)	0.91226 (4)	0.0650 (2)	
S2	0.82255 (5)	0.06276 (5)	0.64335 (3)	0.0633 (2)	
O1	0.51521 (13)	−0.09814 (10)	0.86877 (9)	0.0613 (4)	
O2	0.77147 (14)	−0.03089 (17)	0.87525 (9)	0.0824 (6)	
N1	0.21182 (12)	0.08291 (11)	0.86246 (8)	0.0406 (4)	
N2	0.07989 (15)	0.12137 (13)	0.78125 (9)	0.0525 (5)	
N3	0.55426 (12)	0.08920 (12)	0.84165 (8)	0.0423 (4)	
N4	0.66458 (12)	0.07339 (12)	0.78224 (8)	0.0438 (4)	
C1	0.16656 (17)	0.29982 (16)	0.68388 (11)	0.0506 (5)	
C2	0.1063 (2)	0.2917 (2)	0.62638 (12)	0.0639 (7)	
C3	0.1088 (2)	0.3821 (3)	0.55737 (14)	0.0811 (9)	
C4	0.1708 (3)	0.4788 (2)	0.54542 (14)	0.0822 (9)	
C5	0.2302 (2)	0.4874 (2)	0.60160 (15)	0.0767 (8)	
C6	0.2283 (2)	0.39910 (17)	0.67062 (13)	0.0632 (7)	
C7	0.16624 (16)	0.20488 (15)	0.75640 (10)	0.0457 (5)	
C8	0.24710 (16)	0.18353 (14)	0.80548 (10)	0.0437 (5)	
C9	0.25238 (15)	0.00677 (14)	0.92704 (10)	0.0418 (5)	
C10	0.17980 (17)	−0.08177 (16)	0.95955 (12)	0.0526 (6)	
C11	0.11095 (17)	0.05086 (15)	0.84477 (11)	0.0476 (5)	
C12	0.36795 (15)	0.02720 (16)	0.94737 (10)	0.0443 (5)	
C13	0.48551 (15)	−0.00160 (14)	0.88323 (10)	0.0410 (5)	
C14	0.76563 (16)	0.00525 (18)	0.80555 (11)	0.0526 (6)	
C15	0.86737 (18)	−0.0181 (2)	0.73343 (12)	0.0615 (7)	
C16	0.65776 (15)	0.10149 (14)	0.69704 (10)	0.0415 (5)	
C17	0.56447 (17)	0.03156 (16)	0.68369 (11)	0.0494 (6)	
C18	0.5583 (2)	0.0620 (2)	0.59546 (13)	0.0697 (8)	
C19	0.5257 (3)	0.1911 (3)	0.56671 (16)	0.0926 (10)	
C20	0.6163 (3)	0.2626 (2)	0.58065 (15)	0.0890 (9)	
C21	0.6218 (2)	0.23148 (16)	0.66925 (13)	0.0635 (7)	
S3	0.00085 (5)	0.34118 (5)	0.95204 (3)	0.0644 (2)	
S4	0.80682 (4)	0.45925 (4)	0.68876 (3)	0.0499 (1)	
O3	0.45903 (13)	0.30931 (11)	0.88067 (9)	0.0627 (5)	
O4	0.72609 (13)	0.31913 (13)	0.91780 (8)	0.0676 (5)	
N5	0.16872 (13)	0.48752 (12)	0.89897 (9)	0.0461 (5)	
N6	0.02651 (14)	0.54441 (14)	0.82469 (10)	0.0563 (5)	
N7	0.52947 (12)	0.47506 (11)	0.88527 (8)	0.0395 (4)	
N8	0.64254 (12)	0.45960 (11)	0.82786 (8)	0.0415 (4)	
C22	0.12270 (18)	0.72081 (17)	0.72858 (12)	0.0572 (6)	
C23	0.0572 (2)	0.7221 (2)	0.67113 (13)	0.0662 (7)	
C24	0.0637 (2)	0.8126 (2)	0.60334 (15)	0.0811 (9)	
C25	0.1349 (3)	0.9027 (3)	0.59114 (15)	0.0872 (9)	
C26	0.2000 (2)	0.9046 (2)	0.64714 (17)	0.0863 (9)	
C27	0.1938 (2)	0.81328 (19)	0.71592 (15)	0.0714 (8)	
C28	0.11981 (17)	0.62186 (16)	0.79871 (12)	0.0518 (6)	
C29	0.20755 (16)	0.58894 (15)	0.84390 (11)	0.0494 (6)	
C30	0.20970 (16)	0.40507 (15)	0.96034 (11)	0.0459 (5)	
C31	0.12971 (18)	0.32183 (17)	0.99445 (12)	0.0559 (6)	
C32	0.05979 (16)	0.46679 (16)	0.88438 (12)	0.0508 (6)	
C33	0.33060 (15)	0.41641 (16)	0.97807 (11)	0.0464 (5)	
C34	0.44507 (15)	0.39476 (14)	0.90929 (10)	0.0416 (5)	
C35	0.73393 (15)	0.37844 (15)	0.84930 (11)	0.0456 (5)	
C36	0.84643 (17)	0.37065 (17)	0.77895 (11)	0.0537 (6)	
C37	0.66435 (15)	0.53459 (13)	0.74539 (10)	0.0400 (5)	
C38	0.55215 (17)	0.54151 (16)	0.70950 (11)	0.0498 (6)	
C39	0.5738 (2)	0.6200 (2)	0.62431 (13)	0.0687 (8)	
C40	0.6054 (2)	0.74073 (19)	0.62337 (15)	0.0780 (9)	
C41	0.7171 (2)	0.73238 (17)	0.65896 (13)	0.0642 (7)	
C42	0.69282 (18)	0.65648 (15)	0.74490 (11)	0.0500 (6)	
O5	0.43460 (15)	−0.31518 (11)	0.92938 (9)	0.0647 (5)	
H2	0.06420	0.22620	0.63380	0.0770*	
H3	0.06790	0.37640	0.51920	0.0970*	
H3A	0.52960	0.15720	0.85200	0.0510*	
H4	0.17250	0.53850	0.49920	0.0990*	
H5	0.27230	0.55310	0.59350	0.0920*	
H6	0.26880	0.40640	0.70860	0.0760*	
H8	0.31230	0.22760	0.80130	0.0520*	
H10	0.19230	−0.14110	1.00350	0.0630*	
H12A	0.37230	−0.02150	1.00010	0.0530*	
H12B	0.36380	0.10830	0.95000	0.0530*	
H15A	0.87850	−0.10100	0.73440	0.0740*	
H15B	0.94720	0.00540	0.73440	0.0740*	
H17A	0.48060	0.04730	0.71810	0.0590*	
H17B	0.59000	−0.05120	0.69930	0.0590*	
H18A	0.63970	0.03850	0.56170	0.0840*	
H18B	0.49470	0.01890	0.58990	0.0840*	
H19A	0.43950	0.21250	0.59560	0.1110*	
H19B	0.52940	0.20850	0.50920	0.1110*	
H20A	0.58890	0.34510	0.56550	0.1070*	
H20B	0.70060	0.24870	0.54630	0.1070*	
H21A	0.68380	0.27570	0.67560	0.0760*	
H21B	0.53950	0.25290	0.70310	0.0760*	
H7	0.51340	0.53600	0.90550	0.0470*	
H23	0.00840	0.66110	0.67870	0.0790*	
H24	0.01920	0.81210	0.56580	0.0970*	
H25	0.13970	0.96300	0.54500	0.1050*	
H26	0.24780	0.96650	0.63910	0.1040*	
H27	0.23780	0.81470	0.75350	0.0860*	
H29	0.27820	0.62690	0.83850	0.0590*	
H31	0.14160	0.25940	1.03680	0.0670*	
H33A	0.33810	0.36070	1.02790	0.0560*	
H33B	0.32930	0.49430	0.98620	0.0560*	
H36A	0.86810	0.29010	0.77390	0.0640*	
H36B	0.91910	0.39830	0.78710	0.0640*	
H38A	0.47580	0.57180	0.74450	0.0600*	
H38B	0.53930	0.46360	0.70730	0.0600*	
H39A	0.64310	0.58420	0.58760	0.0830*	
H39B	0.49800	0.62780	0.60520	0.0830*	
H40A	0.62520	0.78650	0.56790	0.0940*	
H40B	0.53200	0.78060	0.65450	0.0940*	
H41A	0.73250	0.81020	0.65990	0.0770*	
H41B	0.79260	0.69940	0.62480	0.0770*	
H42A	0.62140	0.69240	0.78020	0.0600*	
H42B	0.76690	0.65050	0.76550	0.0600*	
H5A	0.459 (2)	−0.2488 (15)	0.9077 (12)	0.0750*	
H5B	0.384 (2)	−0.3144 (19)	0.9756 (10)	0.0750*	

### Atomic displacement parameters (Å^2^)

**Table:**

	*U*^11^	*U*^22^	*U*^33^	*U*^12^	*U*^13^	*U*^23^
S1	0.0629 (3)	0.0560 (3)	0.0770 (4)	−0.0232 (2)	−0.0327 (3)	0.0105 (2)
S2	0.0430 (2)	0.0949 (4)	0.0429 (3)	−0.0046 (2)	0.0006 (2)	−0.0132 (2)
O1	0.0593 (8)	0.0384 (6)	0.0690 (9)	−0.0023 (6)	0.0044 (7)	−0.0063 (6)
O2	0.0527 (8)	0.1431 (15)	0.0474 (8)	0.0145 (9)	−0.0170 (7)	−0.0237 (9)
N1	0.0368 (7)	0.0405 (7)	0.0420 (7)	−0.0023 (5)	−0.0119 (6)	−0.0036 (6)
N2	0.0518 (9)	0.0530 (8)	0.0545 (9)	−0.0033 (7)	−0.0239 (7)	−0.0044 (7)
N3	0.0376 (7)	0.0424 (7)	0.0424 (7)	−0.0032 (6)	−0.0002 (6)	−0.0128 (6)
N4	0.0356 (7)	0.0531 (8)	0.0401 (7)	−0.0056 (6)	−0.0024 (6)	−0.0130 (6)
C1	0.0442 (9)	0.0545 (10)	0.0407 (9)	0.0109 (8)	−0.0059 (7)	−0.0035 (8)
C2	0.0599 (12)	0.0743 (13)	0.0494 (11)	0.0076 (10)	−0.0155 (9)	−0.0060 (10)
C3	0.0772 (15)	0.1069 (19)	0.0469 (12)	0.0196 (14)	−0.0211 (11)	−0.0067 (12)
C4	0.0858 (17)	0.0768 (15)	0.0532 (13)	0.0167 (13)	−0.0061 (12)	0.0121 (11)
C5	0.0836 (16)	0.0564 (12)	0.0642 (14)	0.0056 (11)	−0.0017 (12)	0.0056 (10)
C6	0.0681 (13)	0.0525 (11)	0.0566 (12)	0.0034 (9)	−0.0112 (10)	−0.0011 (9)
C7	0.0420 (9)	0.0455 (9)	0.0447 (9)	0.0026 (7)	−0.0103 (7)	−0.0063 (7)
C8	0.0401 (8)	0.0418 (8)	0.0443 (9)	−0.0037 (7)	−0.0098 (7)	−0.0023 (7)
C9	0.0362 (8)	0.0467 (9)	0.0363 (8)	0.0013 (7)	−0.0081 (6)	−0.0030 (7)
C10	0.0452 (9)	0.0513 (10)	0.0549 (11)	−0.0054 (8)	−0.0166 (8)	0.0043 (8)
C11	0.0452 (9)	0.0448 (9)	0.0539 (10)	−0.0060 (7)	−0.0192 (8)	−0.0048 (8)
C12	0.0349 (8)	0.0588 (10)	0.0346 (8)	−0.0031 (7)	−0.0070 (6)	−0.0053 (7)
C13	0.0359 (8)	0.0446 (9)	0.0378 (8)	−0.0011 (7)	−0.0094 (6)	−0.0028 (7)
C14	0.0363 (9)	0.0787 (12)	0.0448 (10)	−0.0029 (8)	−0.0096 (7)	−0.0198 (9)
C15	0.0402 (9)	0.0950 (15)	0.0507 (11)	0.0047 (9)	−0.0099 (8)	−0.0272 (10)
C16	0.0402 (8)	0.0405 (8)	0.0401 (9)	−0.0058 (7)	−0.0038 (7)	−0.0087 (7)
C17	0.0485 (10)	0.0514 (10)	0.0506 (10)	−0.0054 (8)	−0.0127 (8)	−0.0148 (8)
C18	0.0665 (13)	0.0924 (16)	0.0579 (13)	−0.0055 (12)	−0.0232 (10)	−0.0230 (11)
C19	0.0980 (19)	0.112 (2)	0.0586 (14)	0.0179 (17)	−0.0357 (14)	−0.0017 (14)
C20	0.113 (2)	0.0595 (13)	0.0659 (15)	0.0046 (13)	−0.0101 (14)	0.0143 (11)
C21	0.0802 (14)	0.0389 (9)	0.0608 (12)	−0.0082 (9)	−0.0071 (10)	−0.0029 (8)
S3	0.0473 (3)	0.0729 (3)	0.0725 (3)	−0.0255 (2)	−0.0150 (2)	−0.0040 (3)
S4	0.0475 (2)	0.0523 (2)	0.0451 (2)	0.0038 (2)	−0.0066 (2)	−0.0133 (2)
O3	0.0584 (8)	0.0493 (7)	0.0831 (10)	−0.0109 (6)	−0.0039 (7)	−0.0319 (7)
O4	0.0530 (8)	0.0756 (9)	0.0524 (8)	0.0118 (7)	−0.0103 (6)	0.0098 (7)
N5	0.0353 (7)	0.0495 (8)	0.0531 (9)	−0.0066 (6)	−0.0074 (6)	−0.0136 (7)
N6	0.0440 (8)	0.0646 (10)	0.0600 (10)	−0.0044 (7)	−0.0123 (7)	−0.0148 (8)
N7	0.0353 (7)	0.0365 (7)	0.0453 (8)	−0.0025 (5)	−0.0061 (6)	−0.0120 (6)
N8	0.0345 (7)	0.0412 (7)	0.0433 (7)	0.0005 (5)	−0.0063 (6)	−0.0060 (6)
C22	0.0445 (10)	0.0602 (11)	0.0560 (11)	0.0065 (8)	−0.0009 (8)	−0.0137 (9)
C23	0.0595 (12)	0.0724 (13)	0.0585 (13)	0.0107 (10)	−0.0097 (10)	−0.0160 (10)
C24	0.0741 (15)	0.0959 (18)	0.0574 (14)	0.0163 (14)	−0.0077 (11)	−0.0139 (13)
C25	0.0711 (15)	0.0965 (19)	0.0581 (14)	0.0143 (14)	0.0064 (12)	0.0069 (13)
C26	0.0650 (14)	0.0745 (15)	0.0913 (19)	−0.0087 (12)	0.0080 (14)	0.0007 (13)
C27	0.0578 (12)	0.0701 (13)	0.0724 (14)	−0.0005 (10)	−0.0078 (10)	−0.0044 (11)
C28	0.0431 (9)	0.0535 (10)	0.0553 (11)	0.0015 (8)	−0.0060 (8)	−0.0168 (8)
C29	0.0397 (9)	0.0477 (9)	0.0575 (11)	−0.0054 (7)	−0.0065 (8)	−0.0116 (8)
C30	0.0387 (8)	0.0503 (9)	0.0476 (10)	−0.0081 (7)	−0.0056 (7)	−0.0128 (8)
C31	0.0472 (10)	0.0613 (11)	0.0568 (11)	−0.0165 (8)	−0.0096 (8)	−0.0063 (9)
C32	0.0371 (8)	0.0595 (10)	0.0560 (11)	−0.0091 (8)	−0.0080 (8)	−0.0148 (9)
C33	0.0414 (9)	0.0513 (9)	0.0466 (9)	−0.0100 (7)	−0.0075 (7)	−0.0121 (8)
C34	0.0374 (8)	0.0401 (8)	0.0484 (9)	−0.0041 (6)	−0.0123 (7)	−0.0102 (7)
C35	0.0385 (8)	0.0449 (9)	0.0500 (10)	−0.0003 (7)	−0.0125 (7)	−0.0053 (8)
C36	0.0400 (9)	0.0583 (11)	0.0553 (11)	0.0053 (8)	−0.0097 (8)	−0.0084 (9)
C37	0.0388 (8)	0.0373 (8)	0.0414 (9)	−0.0016 (6)	−0.0085 (7)	−0.0076 (7)
C38	0.0456 (9)	0.0488 (9)	0.0565 (11)	−0.0025 (7)	−0.0192 (8)	−0.0088 (8)
C39	0.0721 (14)	0.0776 (14)	0.0593 (13)	−0.0055 (11)	−0.0335 (11)	−0.0023 (10)
C40	0.0891 (17)	0.0597 (12)	0.0755 (15)	−0.0061 (12)	−0.0310 (13)	0.0124 (11)
C41	0.0710 (13)	0.0423 (10)	0.0718 (14)	−0.0097 (9)	−0.0142 (11)	−0.0013 (9)
C42	0.0513 (10)	0.0418 (9)	0.0566 (11)	−0.0050 (7)	−0.0109 (8)	−0.0133 (8)
O5	0.0816 (10)	0.0369 (6)	0.0617 (9)	−0.0066 (6)	0.0052 (7)	−0.0120 (6)

### Geometric parameters (Å, °)

**Table:**

S1—C10	1.745 (2)	C6—H6	0.9300
S1—C11	1.7293 (19)	C8—H8	0.9300
S2—C15	1.789 (2)	C10—H10	0.9300
S2—C16	1.8411 (18)	C12—H12B	0.9700
S3—C32	1.738 (2)	C12—H12A	0.9700
S3—C31	1.743 (2)	C15—H15B	0.9700
S4—C37	1.8413 (17)	C15—H15A	0.9700
S4—C36	1.7929 (19)	C17—H17B	0.9700
O1—C13	1.214 (2)	C17—H17A	0.9700
O2—C14	1.209 (2)	C18—H18B	0.9700
O3—C34	1.216 (2)	C18—H18A	0.9700
O4—C35	1.218 (2)	C19—H19A	0.9700
O5—H5B	0.848 (18)	C19—H19B	0.9700
O5—H5A	0.833 (19)	C20—H20A	0.9700
N1—C11	1.366 (2)	C20—H20B	0.9700
N1—C8	1.377 (2)	C21—H21A	0.9700
N1—C9	1.395 (2)	C21—H21B	0.9700
N2—C11	1.311 (2)	C22—C23	1.396 (3)
N2—C7	1.384 (2)	C22—C27	1.384 (3)
N3—N4	1.3839 (19)	C22—C28	1.467 (3)
N3—C13	1.345 (2)	C23—C24	1.380 (3)
N4—C16	1.473 (2)	C24—C25	1.362 (4)
N4—C14	1.368 (2)	C25—C26	1.381 (4)
N3—H3A	0.8600	C26—C27	1.397 (4)
N5—C32	1.366 (2)	C28—C29	1.371 (3)
N5—C29	1.379 (2)	C30—C31	1.335 (3)
N5—C30	1.388 (2)	C30—C33	1.485 (3)
N6—C32	1.306 (3)	C33—C34	1.522 (2)
N6—C28	1.388 (3)	C35—C36	1.494 (3)
N7—C34	1.342 (2)	C37—C42	1.526 (2)
N7—N8	1.3845 (19)	C37—C38	1.523 (3)
N8—C37	1.472 (2)	C38—C39	1.525 (3)
N8—C35	1.346 (2)	C39—C40	1.523 (3)
N7—H7	0.8600	C40—C41	1.512 (3)
C1—C2	1.389 (3)	C41—C42	1.524 (3)
C1—C7	1.471 (3)	C23—H23	0.9300
C1—C6	1.388 (3)	C24—H24	0.9300
C2—C3	1.397 (3)	C25—H25	0.9300
C3—C4	1.368 (4)	C26—H26	0.9300
C4—C5	1.363 (4)	C27—H27	0.9300
C5—C6	1.385 (3)	C29—H29	0.9300
C7—C8	1.364 (3)	C31—H31	0.9300
C9—C10	1.330 (3)	C33—H33A	0.9700
C9—C12	1.486 (2)	C33—H33B	0.9700
C12—C13	1.517 (2)	C36—H36B	0.9700
C14—C15	1.491 (3)	C36—H36A	0.9700
C16—C17	1.519 (3)	C38—H38A	0.9700
C16—C21	1.521 (3)	C38—H38B	0.9700
C17—C18	1.522 (3)	C39—H39A	0.9700
C18—C19	1.507 (4)	C39—H39B	0.9700
C19—C20	1.513 (5)	C40—H40B	0.9700
C20—C21	1.525 (3)	C40—H40A	0.9700
C2—H2	0.9300	C41—H41A	0.9700
C3—H3	0.9300	C41—H41B	0.9700
C4—H4	0.9300	C42—H42B	0.9700
C5—H5	0.9300	C42—H42A	0.9700
			
S1···O2^i^	3.3487 (17)	C6···H8	2.9200
S1···N1	2.5482 (15)	C8···H12B	3.0400
S1···S1^ii^	3.7967 (8)	C8···H17A	3.0600
S1···S3^ii^	3.4544 (8)	C8···H6	2.7600
S1···C10^ii^	3.581 (2)	C13···H12A^v^	3.0000
S1···C31^ii^	3.608 (2)	C13···H5A	2.906 (19)
S2···N4	2.5890 (14)	C13···H17A	2.8300
S2···C25^iii^	3.655 (3)	C17···H41A^vi^	3.0200
S3···C10^ii^	3.685 (2)	C21···H3A	3.0200
S3···O4^i^	3.3109 (16)	C21···H38B	2.9500
S3···N5	2.5507 (16)	C23···H15A^viii^	2.8600
S3···S1^ii^	3.4544 (8)	C23···H5	3.1000
S4···C3^iv^	3.610 (3)	C24···H15A^viii^	2.9000
S4···N8	2.6008 (14)	C26···H15B^viii^	3.0000
S4···C2^iv^	3.615 (2)	C27···H29	2.9100
S1···H31^ii^	3.1300	C29···H27	2.7600
S2···H18A	2.8700	C29···H38A	2.9800
S2···H20B	2.8400	C29···H33B	3.0600
S3···H36B^i^	3.1600	C32···H36B^i^	2.9400
S4···H41B	2.7800	C34···H38A	3.0800
S4···H39A	2.8700	C34···H5B^v^	3.03 (2)
S4···H21A	2.8300	C35···H5B^v^	2.934 (17)
O1···N4	2.686 (2)	C39···H19B^ix^	3.0500
O1···O2	3.092 (2)	C40···H19B^ix^	3.0000
O1···C14	2.996 (2)	C40···H17B^vii^	3.0600
O1···C17	3.159 (2)	C41···H17B^vii^	2.8900
O1···O5	2.7097 (19)	C42···H7	3.1000
O2···N3	2.724 (2)	H2···N2	2.6200
O2···S1^iv^	3.3487 (17)	H3A···C21	3.0200
O2···O1	3.092 (2)	H3A···O3	1.9800
O2···C13	3.082 (2)	H3A···H12B	2.1600
O2···C12^v^	3.074 (2)	H3A···H21B	2.5600
O3···C35	3.122 (2)	H5···C23	3.1000
O3···N3	2.841 (2)	H5A···O1	1.880 (19)
O3···N8	2.711 (2)	H5A···C13	2.906 (19)
O3···O4	3.215 (2)	H5A···H7^vi^	2.5400
O4···C10^v^	3.306 (2)	H5B···C34^v^	3.03 (2)
O4···O5^v^	2.764 (2)	H5B···C35^v^	2.934 (17)
O4···N7	2.713 (2)	H5B···H7^vi^	2.4600
O4···C34	3.121 (2)	H5B···H33B^vi^	2.3900
O4···S3^iv^	3.3109 (16)	H5B···O4^v^	1.917 (18)
O4···O3	3.215 (2)	H6···C8	2.7600
O5···C34^v^	3.331 (2)	H6···H8	2.4000
O5···C33^vi^	3.376 (2)	H7···H5B^vii^	2.4600
O5···N7^vi^	2.7617 (19)	H7···H5A^vii^	2.5400
O5···C33^v^	3.349 (2)	H7···C42	3.1000
O5···O1	2.7097 (19)	H7···O5^vii^	1.9400
O5···O4^v^	2.764 (2)	H7···H33B	2.1700
O1···H17A	2.8600	H8···H6	2.4000
O1···H5A	1.880 (19)	H8···O3	2.8300
O1···H17B	2.7900	H8···C6	2.9200
O2···H12A^v^	2.4800	H10···O4^v^	2.4100
O3···H8	2.8300	H10···H12A	2.5900
O3···H3A	1.9800	H12A···H10	2.5900
O3···H12B	2.6200	H12A···C13^v^	3.0000
O4···H10^v^	2.4100	H12A···O2^v^	2.4800
O4···H5B^v^	1.917 (18)	H12B···O3	2.6200
O5···H33B^vi^	2.5400	H12B···C8	3.0400
O5···H42A^vi^	2.8300	H12B···H3A	2.1600
O5···H29^vi^	2.9200	H15A···C24^iii^	2.9000
O5···H33A^v^	2.7500	H15A···C23^iii^	2.8600
O5···H7^vi^	1.9400	H15B···C26^iii^	3.0000
N1···S1	2.5482 (15)	H15B···N2^iv^	2.5700
N1···N2	2.237 (2)	H17A···H21B	2.5500
N2···N1	2.237 (2)	H17A···C13	2.8300
N3···O2	2.724 (2)	H17A···O1	2.8600
N3···O3	2.841 (2)	H17A···N3	2.7100
N4···S2	2.5890 (14)	H17A···C8	3.0600
N4···O1	2.686 (2)	H17B···C40^vi^	3.0600
N5···N6	2.237 (2)	H17B···C41^vi^	2.8900
N5···S3	2.5507 (16)	H17B···H40B^vi^	2.5500
N6···C36^i^	3.448 (3)	H17B···O1	2.7900
N6···N5	2.237 (2)	H17B···H41A^vi^	2.2000
N7···O4	2.713 (2)	H18A···S2	2.8700
N7···O5^vii^	2.7617 (19)	H19B···C39^ix^	3.0500
N8···S4	2.6008 (14)	H19B···H39B^ix^	2.4700
N8···O3	2.711 (2)	H19B···H40A^ix^	2.4500
N2···H36A^i^	2.8400	H19B···C40^ix^	3.0000
N2···H15B^i^	2.5700	H20B···S2	2.8400
N2···H2	2.6200	H21A···S4	2.8300
N3···H17A	2.7100	H21B···N3	2.7300
N3···H21B	2.7300	H21B···H17A	2.5500
N6···H36B^i^	2.5600	H21B···H38B	2.5200
N6···H23	2.6400	H21B···H3A	2.5600
N7···H42A	2.9000	H23···N6	2.6400
N7···H38A	2.6400	H27···C29	2.7600
C1···C36^i^	3.521 (3)	H27···H29	2.4000
C2···S4^i^	3.615 (2)	H29···C27	2.9100
C2···C36^i^	3.519 (3)	H29···H27	2.4000
C3···S4^i^	3.610 (3)	H29···H38A	2.4400
C5···C23	3.417 (3)	H29···O5^vii^	2.9200
C8···C13	3.508 (2)	H31···S1^ii^	3.1300
C10···S1^ii^	3.581 (2)	H31···H33A	2.5900
C10···O4^v^	3.306 (2)	H33A···H31	2.5900
C10···S3^ii^	3.685 (2)	H33A···O5^v^	2.7500
C12···O2^v^	3.074 (2)	H33B···O5^vii^	2.5400
C13···C8	3.508 (2)	H33B···C29	3.0600
C13···C17	3.306 (2)	H33B···H5B^vii^	2.3900
C13···O2	3.082 (2)	H33B···H7	2.1700
C14···O1	2.996 (2)	H36A···N2^iv^	2.8400
C15···C24^iii^	3.488 (3)	H36B···C1^iv^	3.0600
C15···C25^iii^	3.465 (4)	H36B···S3^iv^	3.1600
C17···C13	3.306 (2)	H36B···N6^iv^	2.5600
C17···O1	3.159 (2)	H36B···C32^iv^	2.9400
C23···C5	3.417 (3)	H38A···N7	2.6400
C24···C15^viii^	3.488 (3)	H38A···C29	2.9800
C25···C15^viii^	3.465 (4)	H38A···H29	2.4400
C25···S2^viii^	3.655 (3)	H38A···C34	3.0800
C29···C34	3.458 (3)	H38B···C21	2.9500
C31···S1^ii^	3.608 (2)	H38B···H21B	2.5200
C33···O5^vii^	3.376 (2)	H39A···C4^ix^	2.8200
C33···O5^v^	3.349 (2)	H39A···S4	2.8700
C34···C38	3.457 (2)	H39B···H19B^ix^	2.4700
C34···O5^v^	3.331 (2)	H40A···H19B^ix^	2.4500
C34···O4	3.121 (2)	H40B···H17B^vii^	2.5500
C34···C29	3.458 (3)	H40B···H42A	2.5900
C35···O3	3.122 (2)	H41A···C17^vii^	3.0200
C36···C1^iv^	3.521 (3)	H41A···H17B^vii^	2.2000
C36···C2^iv^	3.519 (3)	H41B···S4	2.7800
C36···N6^iv^	3.448 (3)	H42A···N7	2.9000
C38···C34	3.457 (2)	H42A···H40B	2.5900
C1···H36B^i^	3.0600	H42A···O5^vii^	2.8300
C4···H39A^ix^	2.8200		
			
C10—S1—C11	89.76 (9)	C18—C19—H19B	109.00
C15—S2—C16	94.55 (9)	C18—C19—H19A	109.00
C31—S3—C32	89.81 (10)	H20A—C20—H20B	108.00
C36—S4—C37	93.69 (8)	C19—C20—H20A	109.00
H5A—O5—H5B	110 (2)	C21—C20—H20B	109.00
C9—N1—C11	115.26 (14)	C21—C20—H20A	109.00
C8—N1—C11	106.06 (14)	C19—C20—H20B	109.00
C8—N1—C9	138.60 (15)	H21A—C21—H21B	108.00
C7—N2—C11	103.65 (16)	C16—C21—H21A	109.00
N4—N3—C13	120.15 (14)	C16—C21—H21B	109.00
C14—N4—C16	121.05 (14)	C20—C21—H21B	109.00
N3—N4—C16	118.40 (13)	C20—C21—H21A	109.00
N3—N4—C14	118.31 (13)	C27—C22—C28	121.11 (19)
C13—N3—H3A	120.00	C23—C22—C28	120.73 (19)
N4—N3—H3A	120.00	C23—C22—C27	118.1 (2)
C29—N5—C30	138.38 (16)	C22—C23—C24	121.0 (2)
C30—N5—C32	115.52 (15)	C23—C24—C25	120.4 (2)
C29—N5—C32	106.09 (15)	C24—C25—C26	120.1 (3)
C28—N6—C32	103.80 (16)	C25—C26—C27	119.9 (2)
N8—N7—C34	119.88 (13)	C22—C27—C26	120.6 (2)
N7—N8—C37	120.22 (13)	C22—C28—C29	126.78 (18)
N7—N8—C35	119.23 (13)	N6—C28—C29	111.15 (17)
C35—N8—C37	120.50 (14)	N6—C28—C22	121.99 (18)
N8—N7—H7	120.00	N5—C29—C28	105.34 (16)
C34—N7—H7	120.00	N5—C30—C33	119.68 (16)
C2—C1—C7	120.74 (18)	C31—C30—C33	129.22 (18)
C6—C1—C7	120.96 (18)	N5—C30—C31	111.08 (17)
C2—C1—C6	118.30 (18)	S3—C31—C30	113.62 (15)
C1—C2—C3	120.0 (2)	N5—C32—N6	113.62 (17)
C2—C3—C4	120.7 (2)	S3—C32—N5	109.94 (14)
C3—C4—C5	119.7 (2)	S3—C32—N6	136.44 (15)
C4—C5—C6	120.6 (2)	C30—C33—C34	111.58 (15)
C1—C6—C5	120.8 (2)	N7—C34—C33	114.61 (15)
N2—C7—C1	121.12 (16)	O3—C34—C33	121.81 (16)
C1—C7—C8	127.44 (17)	O3—C34—N7	123.55 (16)
N2—C7—C8	111.42 (15)	N8—C35—C36	111.73 (15)
N1—C8—C7	105.50 (15)	O4—C35—N8	124.10 (17)
C10—C9—C12	129.00 (17)	O4—C35—C36	124.17 (17)
N1—C9—C12	120.05 (15)	S4—C36—C35	108.23 (13)
N1—C9—C10	110.83 (16)	N8—C37—C38	111.38 (14)
S1—C10—C9	113.87 (15)	S4—C37—C38	110.84 (12)
S1—C11—N2	136.35 (16)	S4—C37—C42	109.67 (12)
S1—C11—N1	110.25 (13)	S4—C37—N8	102.87 (11)
N1—C11—N2	113.38 (16)	N8—C37—C42	110.98 (13)
C9—C12—C13	109.81 (14)	C38—C37—C42	110.85 (14)
O1—C13—N3	121.85 (16)	C37—C38—C39	111.58 (16)
N3—C13—C12	114.82 (15)	C38—C39—C40	111.30 (18)
O1—C13—C12	123.31 (16)	C39—C40—C41	111.23 (19)
O2—C14—N4	124.47 (18)	C40—C41—C42	111.33 (18)
N4—C14—C15	110.82 (16)	C37—C42—C41	110.31 (15)
O2—C14—C15	124.72 (19)	C24—C23—H23	120.00
S2—C15—C14	108.78 (15)	C22—C23—H23	119.00
C17—C16—C21	109.66 (15)	C23—C24—H24	120.00
N4—C16—C17	112.39 (14)	C25—C24—H24	120.00
N4—C16—C21	110.48 (14)	C26—C25—H25	120.00
S2—C16—C17	111.81 (12)	C24—C25—H25	120.00
S2—C16—N4	102.18 (11)	C25—C26—H26	120.00
S2—C16—C21	110.13 (13)	C27—C26—H26	120.00
C16—C17—C18	111.68 (16)	C26—C27—H27	120.00
C17—C18—C19	111.47 (19)	C22—C27—H27	120.00
C18—C19—C20	111.3 (2)	N5—C29—H29	127.00
C19—C20—C21	111.4 (2)	C28—C29—H29	127.00
C16—C21—C20	111.24 (17)	C30—C31—H31	123.00
C1—C2—H2	120.00	S3—C31—H31	123.00
C3—C2—H2	120.00	C30—C33—H33A	109.00
C4—C3—H3	120.00	C34—C33—H33A	109.00
C2—C3—H3	120.00	C34—C33—H33B	109.00
C3—C4—H4	120.00	C30—C33—H33B	109.00
C5—C4—H4	120.00	H33A—C33—H33B	108.00
C6—C5—H5	120.00	S4—C36—H36B	110.00
C4—C5—H5	120.00	C35—C36—H36A	110.00
C5—C6—H6	120.00	C35—C36—H36B	110.00
C1—C6—H6	120.00	H36A—C36—H36B	108.00
N1—C8—H8	127.00	S4—C36—H36A	110.00
C7—C8—H8	127.00	C37—C38—H38B	109.00
C9—C10—H10	123.00	C39—C38—H38A	109.00
S1—C10—H10	123.00	C37—C38—H38A	109.00
C13—C12—H12B	110.00	H38A—C38—H38B	108.00
C9—C12—H12B	110.00	C39—C38—H38B	109.00
H12A—C12—H12B	108.00	C38—C39—H39A	109.00
C13—C12—H12A	110.00	C38—C39—H39B	109.00
C9—C12—H12A	110.00	C40—C39—H39B	109.00
S2—C15—H15B	110.00	H39A—C39—H39B	108.00
C14—C15—H15A	110.00	C40—C39—H39A	109.00
C14—C15—H15B	110.00	C39—C40—H40B	109.00
H15A—C15—H15B	108.00	C41—C40—H40A	109.00
S2—C15—H15A	110.00	C41—C40—H40B	109.00
C18—C17—H17B	109.00	H40A—C40—H40B	108.00
C16—C17—H17B	109.00	C39—C40—H40A	109.00
H17A—C17—H17B	108.00	C40—C41—H41B	109.00
C18—C17—H17A	109.00	C42—C41—H41A	109.00
C16—C17—H17A	109.00	C40—C41—H41A	109.00
C19—C18—H18A	109.00	H41A—C41—H41B	108.00
C17—C18—H18B	109.00	C42—C41—H41B	109.00
C17—C18—H18A	109.00	C37—C42—H42A	110.00
C19—C18—H18B	109.00	C37—C42—H42B	110.00
H18A—C18—H18B	108.00	C41—C42—H42B	110.00
C20—C19—H19A	109.00	H42A—C42—H42B	108.00
C20—C19—H19B	109.00	C41—C42—H42A	110.00
H19A—C19—H19B	108.00		
			
C10—S1—C11—N2	176.9 (2)	C37—N8—C35—O4	174.60 (17)
C10—S1—C11—N1	−1.42 (14)	C37—N8—C35—C36	−5.0 (2)
C11—S1—C10—C9	0.62 (16)	N7—N8—C37—S4	−167.85 (11)
C15—S2—C16—N4	14.50 (13)	C6—C1—C7—N2	163.05 (18)
C16—S2—C15—C14	−11.71 (16)	C2—C1—C7—C8	160.2 (2)
C15—S2—C16—C17	−105.91 (14)	C2—C1—C7—N2	−17.7 (3)
C15—S2—C16—C21	131.92 (14)	C7—C1—C6—C5	178.9 (2)
C31—S3—C32—N6	178.7 (2)	C6—C1—C2—C3	0.0 (3)
C32—S3—C31—C30	0.70 (16)	C7—C1—C2—C3	−179.3 (2)
C31—S3—C32—N5	−1.50 (15)	C2—C1—C6—C5	−0.3 (3)
C37—S4—C36—C35	14.05 (14)	C6—C1—C7—C8	−19.0 (3)
C36—S4—C37—C42	102.61 (13)	C1—C2—C3—C4	0.4 (4)
C36—S4—C37—N8	−15.53 (12)	C2—C3—C4—C5	−0.4 (4)
C36—S4—C37—C38	−134.68 (13)	C3—C4—C5—C6	0.0 (4)
C9—N1—C11—N2	−176.73 (15)	C4—C5—C6—C1	0.3 (4)
C8—N1—C11—S1	179.35 (12)	N2—C7—C8—N1	0.7 (2)
C11—N1—C9—C12	174.83 (15)	C1—C7—C8—N1	−177.46 (17)
C11—N1—C8—C7	−0.77 (18)	C10—C9—C12—C13	103.4 (2)
C8—N1—C9—C10	−177.71 (19)	C12—C9—C10—S1	−175.59 (15)
C9—N1—C11—S1	1.97 (19)	N1—C9—C12—C13	−72.2 (2)
C8—N1—C9—C12	−1.4 (3)	N1—C9—C10—S1	0.4 (2)
C8—N1—C11—N2	0.7 (2)	C9—C12—C13—N3	117.53 (17)
C11—N1—C9—C10	−1.5 (2)	C9—C12—C13—O1	−61.1 (2)
C9—N1—C8—C7	175.65 (18)	N4—C14—C15—S2	4.7 (2)
C11—N2—C7—C1	177.98 (17)	O2—C14—C15—S2	−175.19 (19)
C11—N2—C7—C8	−0.3 (2)	N4—C16—C21—C20	179.11 (19)
C7—N2—C11—N1	−0.2 (2)	N4—C16—C17—C18	179.80 (15)
C7—N2—C11—S1	−178.47 (17)	C17—C16—C21—C20	−56.5 (2)
N4—N3—C13—O1	−2.2 (2)	S2—C16—C21—C20	67.0 (2)
N4—N3—C13—C12	179.16 (14)	S2—C16—C17—C18	−65.96 (18)
C13—N3—N4—C16	96.51 (18)	C21—C16—C17—C18	56.5 (2)
C13—N3—N4—C14	−66.7 (2)	C16—C17—C18—C19	−56.0 (3)
C16—N4—C14—O2	−172.19 (19)	C17—C18—C19—C20	54.4 (3)
N3—N4—C14—C15	170.62 (16)	C18—C19—C20—C21	−54.6 (3)
N3—N4—C16—C17	−58.36 (19)	C19—C20—C21—C16	56.1 (3)
C14—N4—C16—C21	−132.82 (18)	C23—C22—C28—C29	157.0 (2)
C14—N4—C16—C17	104.34 (19)	C27—C22—C28—C29	−20.6 (3)
C14—N4—C16—S2	−15.66 (19)	C27—C22—C28—N6	163.0 (2)
N3—N4—C14—O2	−9.5 (3)	C27—C22—C23—C24	0.4 (3)
N3—N4—C16—C21	64.5 (2)	C23—C22—C28—N6	−19.3 (3)
N3—N4—C16—S2	−178.36 (12)	C23—C22—C27—C26	−0.4 (3)
C16—N4—C14—C15	7.9 (2)	C28—C22—C27—C26	177.3 (2)
C32—N5—C30—C33	176.91 (16)	C28—C22—C23—C24	−177.4 (2)
C30—N5—C32—N6	−178.10 (16)	C22—C23—C24—C25	0.2 (4)
C29—N5—C32—S3	−179.06 (12)	C23—C24—C25—C26	−0.7 (4)
C30—N5—C32—S3	2.0 (2)	C24—C25—C26—C27	0.7 (4)
C29—N5—C32—N6	0.8 (2)	C25—C26—C27—C22	−0.1 (4)
C29—N5—C30—C33	−1.5 (3)	N6—C28—C29—N5	0.7 (2)
C32—N5—C30—C31	−1.5 (2)	C22—C28—C29—N5	−176.02 (18)
C32—N5—C29—C28	−0.9 (2)	C31—C30—C33—C34	109.4 (2)
C30—N5—C29—C28	177.7 (2)	N5—C30—C31—S3	0.3 (2)
C29—N5—C30—C31	−180.0 (2)	N5—C30—C33—C34	−68.7 (2)
C28—N6—C32—N5	−0.4 (2)	C33—C30—C31—S3	−177.96 (16)
C32—N6—C28—C22	176.68 (18)	C30—C33—C34—N7	132.25 (16)
C28—N6—C32—S3	179.43 (18)	C30—C33—C34—O3	−49.7 (2)
C32—N6—C28—C29	−0.2 (2)	O4—C35—C36—S4	172.38 (16)
N8—N7—C34—O3	−3.4 (3)	N8—C35—C36—S4	−8.03 (19)
C34—N7—N8—C37	109.47 (17)	S4—C37—C38—C39	−66.61 (18)
N8—N7—C34—C33	174.63 (14)	N8—C37—C38—C39	179.50 (15)
C34—N7—N8—C35	−73.1 (2)	C42—C37—C38—C39	55.4 (2)
N7—N8—C35—C36	177.60 (14)	S4—C37—C42—C41	66.18 (18)
N7—N8—C35—O4	−2.8 (3)	N8—C37—C42—C41	179.15 (15)
C35—N8—C37—S4	14.76 (18)	C38—C37—C42—C41	−56.5 (2)
C35—N8—C37—C38	133.53 (16)	C37—C38—C39—C40	−54.3 (2)
N7—N8—C37—C38	−49.08 (19)	C38—C39—C40—C41	54.6 (2)
C35—N8—C37—C42	−102.45 (18)	C39—C40—C41—C42	−56.4 (2)
N7—N8—C37—C42	74.93 (19)	C40—C41—C42—C37	57.3 (2)

Symmetry codes: (i) *x*−1, *y*, *z*; (ii) −*x*, −*y*, −*z*+2; (iii) *x*+1, *y*−1, *z*; (iv) *x*+1, *y*, *z*; (v) −*x*+1, −*y*, −*z*+2; (vi) *x*, *y*−1, *z*; (vii) *x*, *y*+1, *z*; (viii) *x*−1, *y*+1, *z*; (ix) −*x*+1, −*y*+1, −*z*+1.

### Hydrogen-bond geometry (Å, °)

**Table:**

*D*—H···*A*	*D*—H	H···*A*	*D*···*A*	*D*—H···*A*
N3—H3A···O3	0.86	1.98	2.841 (2)	175
O5—H5A···O1	0.833 (19)	1.880 (19)	2.7097 (19)	174.4 (18)
O5—H5B···O4^v^	0.848 (18)	1.917 (18)	2.764 (2)	177 (2)
N7—H7···O5^vii^	0.86	1.94	2.7617 (19)	160
C10—H10···O4^v^	0.93	2.41	3.306 (2)	161
C12—H12A···O2^v^	0.97	2.48	3.074 (2)	120
C15—H15B···N2^iv^	0.97	2.57	3.462 (3)	153
C18—H18A···S2	0.97	2.87	3.255 (2)	105
C20—H20B···S2	0.97	2.84	3.227 (3)	105
C21—H21A···S4	0.97	2.83	3.768 (2)	163
C33—H33B···O5^vii^	0.97	2.54	3.376 (2)	144
C36—H36B···N6^iv^	0.97	2.56	3.448 (3)	153
C39—H39A···S4	0.97	2.87	3.246 (2)	104
C41—H41B···S4	0.97	2.78	3.194 (2)	106

Symmetry codes: (v) −*x*+1, −*y*, −*z*+2; (vii) *x*, *y*+1, *z*; (iv) *x*+1, *y*, *z*.

## References

[bb1] Akkurt, M., Yalçın, Ş. P., Gürsoy, E., Güzeldemirci, N. U. & Büyükgüngör, O. (2007). *Acta Cryst.* E**63**, o3103.

[bb2] Akkurt, M., Yıldırım, S. Ö., Ur, F., Cesur, Z., Cesur, N. & Büyükgüngör, O. (2005). *Acta Cryst.* E**61**, o718–o720.

[bb3] Allen, F. H., Kennard, O., Watson, D. G., Brammer, L., Orpen, A. G. & Taylor, R. (1987). *J. Chem. Soc. Perkin Trans. 2*, pp. S1–19.

[bb4] Altomare, A., Burla, M. C., Camalli, M., Cascarano, G. L., Giacovazzo, C., Guagliardi, A., Moliterni, A. G. G., Polidori, G. & Spagna, R. (1999). *J. Appl. Cryst.* **32**, 115–119.

[bb5] Amarouch, H., Loiseau, P. R., Bonnafous, M., Caujolle, R., Payard, M., Loiseau, P. M., Bories, C. & Gayral, P. (1988). *Farmaco Ed. Sci.* **43**, 421–437. 3220127

[bb6] Andreani, A., Leoni, A., Morigi, R., Bossa, R., Chiericozzi, M. & Galatulas, I. (1998). *Arzneim. Forsch. Drug. Res.* **48**, 232–235. 9553678

[bb7] Cremer, D. & Pople, J. A. (1975). *J. Am. Chem. Soc.* **97**, 1354–1358.

[bb8] Devlin, J. P. & Hargrave, K. D. (1989). *Tetrahedron*, **45**, 4327–4369.

[bb9] Farrugia, L. J. (1997). *J. Appl. Cryst.* **30**, 565.

[bb10] Farrugia, L. J. (1999). *J. Appl. Cryst.* **32**, 837–838.

[bb11] Gürsoy, E. & Ulusoy Güzeldemirci, N. (2007). *Eur. J. Med. Chem.* **42**, 320–326. 10.1016/j.ejmech.2006.10.012 17145120

[bb12] Öztürk Yıldırım, S., Akkurt, M., Ur, F., Cesur, Z., Cesur, N. & Büyükgüngör, O. (2005). *Acta Cryst.* E**61**, o892–o894.

[bb13] Öztürk Yıldırım, S., Akkurt, M., Ur, F., Cesur, Z., Cesur, N. & Heinemann, F. W. (2005). *Acta Cryst.* E**61**, o2357–o2359.

[bb14] Sheldrick, G. M. (2008). *Acta Cryst.* A**64**, 112–122. 10.1107/S0108767307043930 18156677

[bb15] Srimanth, K., Rao, V. R. & Krishna, D. R. (2002). *Arzneim. Forsch. Drug. Res.* **52**, 388–392. 10.1055/s-0031-1299903 12087925

[bb16] Stoe & Cie (2002). *X-AREA* and *X-RED32* Stoe & Cie, Darmstadt, Germany.

[bb17] Ulusoy, N. (2002). *Arzneim. Forsch. Drug. Res.* **52**, 565–571.

[bb18] Ur, F., Cesur, N., Birteksöz, S. & Ötük, G. (2004). *Arzneim. Forsch. Drug. Res.* **54**, 125–129. 10.1055/s-0031-1296947 15038463

